# Genetic characteristics, antimicrobial resistance, and prevalence of *Arcobacter* spp. isolated from various sources in Shenzhen, China

**DOI:** 10.3389/fmicb.2022.1004224

**Published:** 2022-12-01

**Authors:** Yanping Ma, Changyan Ju, Guilan Zhou, Muhua Yu, Hui Chen, Jiaoming He, Maojun Zhang, Yongxiang Duan

**Affiliations:** ^1^Nanshan Center for Disease Control and Prevention, Shenzhen, China; ^2^State Key Laboratory of Infectious Disease Prevention and Control, Collaborative Innovation Center for Diagnosis and Treatment of Infectious Diseases, National Institute for Communicable Disease Control and Prevention, Chinese Center for Disease Control and Prevention, Beijing, China

**Keywords:** *Arcobacter*, whole genome sequencing, antibiotic resistance, phylogenomic analysis, zoonotic pathogen

## Abstract

*Arcobacter* spp. is a globally emerging zoonotic and foodborne pathogen. However, little is known about its prevalence and antimicrobial resistance in China. To investigate the prevalence of *Arcobacter* spp. isolated from various sources, 396 samples were collected from human feces, chicken cecum, and food specimens including chicken meat, beef, pork, lettuce, and seafood. *Arcobacter* spp. was isolated by the membrane filtration method. For 92 strains, the agar dilution method and next-generation sequencing were used to investigate their antimicrobial resistance and to obtain whole genome data, respectively. The virulence factor database (VFDB) was queried to identify virulence genes. ResFinder and the Comprehensive Antibiotic Resistance Database (CARD) were used to predict resistance genes. A phylogenetic tree was constructed using the maximum likelihood (ML) method with core single-nucleotide polymorphisms (SNPs). We found that 27.5% of the samples (*n* = 109) were positive for *Arcobacter* spp., comprising *Arcobacter butzleri* (53.0%), *Arcobacter cryaerophilus* (39.6%), and *Arcobacter skirrowii* (7.4%). Chicken meat had the highest prevalence (81.2%), followed by seafood (51.9%), pork (43.3%), beef (36.7%), lettuce (35.5%), chicken cecum (8%), and human fecal samples (0%, 0/159). Antimicrobial susceptibility tests revealed that 51 *A. butzleri* and 40 *A. cryaerophilus* strains were resistant to streptomycin (98.1, 70%), clindamycin (94.1, 90%), tetracycline (64.7, 52.5%), azithromycin (43.1%, 15%), nalidixic acid (33.4, 35%), and ciprofloxacin (31.3, 35%) but were susceptible to erythromycin, gentamicin, chloramphenicol, telithromycin, and clindamycin (≤10%). *A. skirrowii* was sensitive to all experimental antibiotics. The virulence factors *tly*A, *mviN, cj1349, ciaB*, and *pldA* were carried by all *Arcobacter* spp. strains at 100%, and the following percentages were *cadF* (95.7%), *iroE* (23.9%), *hecB (*2.2%*), hecA*, and *irgA* (1.1%). Only one *A. butzleri* strain (F061-2G) carried a macrolide resistance gene (*ereA*). One *A. butzleri* and one *A. cryaerophilus* harbored resistance island gene clusters, which were isolated from pork and chicken. Phylogenetic tree analysis revealed that *A. butzleri, A. cryaerophilus*, and *A. skirrowii* were separated from each other. To our knowledge, this is the first report of the isolation of *Arcobacter* spp. from vegetables and seafood in China. The resistance island gene cluster found in pork and chicken meat and the presence of virulence factors could be a potential risk to human health.

## Introduction

*Arcobacter* is a globally emerging foodborne pathogen causing diarrhea, enteritis, and bacteremia in humans and diarrhea, mastitis, and abortion in animals (Ramees et al., 2017; Zambri et al., 2019; Chieffi et al., 2020; Khodamoradi and Abiri, 2020). Humans are mainly infected with *Arcobacter via* the consumption of contaminated food and water (Collado and Figueras, 2011; Šilha et al., 2019). The main strains causing diseases in humans are *A. butzleri, A. cryaerophilus*, and *A. skirrowii* (Van den Abeele et al., 2014; Ferreira et al., 2019).

*Arcobacter*-infected poultry is considered the major source of infection (Jribi et al., 2020; Khodamoradi and Abiri, 2020). The prevalence of *Arcobacter* in broiler chickens was reported as 26.0% (26/100) in Iran (Khodamoradi and Abiri, 2020), 55.7% (54/97) in the south of Chile (Vidal-Veuthey et al., 2021), and 73.33% (44/60) in Beijing, China (Wang et al., 2016). Moreover, *Arcobacter* is also found in beef, pork, vegetables, and seafood (Mottola et al., 2016, 2021; Córdoba-Calderón et al., 2017; Kim et al., 2019; Zhang et al., 2019; Jasim et al., 2021), which represent possible transmission sources to humans. However, there have been a few reports regarding *Arcobacter* isolated from various sources in China.

Although most *Arcobacter* infections are self-limited, antibiotic treatment is required for severe clinical infections (Ferreira et al., 2019). This treatment usually includes quinolones, tetracyclines, macrolides, and β-lactamase (Figueras et al., 2014). However, high resistance rates of *Arcobacter* isolates to quinolones and macrolides have been reported (Ferreira et al., 2016; Dekker et al., 2019; Jribi et al., 2020).

Currently, our knowledge is limited concerning the pathogenic mechanisms and virulence features of *Arcobacter* strains (Oliveira et al., 2018; Parisi et al., 2019; Šilha et al., 2019). It was found that 10 potential virulence genes (*iroE, irgA, tlyA, pldA, mviN, hecB, hecA, ciaB, cj1349, and cadF*) were considered important for the virulence of this pathogen (Miller et al., 2007; Rathlavath et al., 2017; Kietsiri et al., 2021). Different virulence genes have different effects on disease (Kietsiri et al., 2021). The existence of virulence factors in *Arcobacter* spp. isolated from food could threaten human health.

This study aimed to determine the prevalence of *Arcobacter* spp. in various sources in Shenzhen, China, and to identify the virulence and antibiotic resistance profiles of *Arcobacter* spp. using whole genome sequencing (WGS). Furthermore, we aimed to determine the minimum inhibitory concentrations (MICs) of 11 common antibiotics to identify the most appropriate and effective treatment for *Arcobacter* infections.

## Methods

### Sample collection

Between June and September 2019, 159 fecal samples were collected from adult patients with diarrhea at the top three local hospitals. In this study, informed consent was obtained from each adult patient with diarrhea. Patients >16 years of age and who experienced acute diarrhea three times or more in the previous 24 h were included. Meanwhile, a collection of 237 samples from chicken meat (*n* = 69), beef (*n* = 30), pork (*n* = 30), lettuce (*n* = 31), and seafood (*n* = 27) were purchased from two retail markets in the Nanshan center; chicken cecum samples (*n* = 50) were collected from a poultry wholesale market.

Fecal samples (~0.5 g each) were collected into the Cary-Blair medium, and food samples (~250 g each) were placed in a sterile plastic bag, and all samples were transported to the laboratory at 4°C within 4 h for bacterial isolation.

### Bacterial culture, isolation, and identification

*Arcobacter* was isolated by an *Arcobacter* isolation kit using the enrichment and membrane filter method (ZC-ARCO-001, Qingdao Sinova Biotechnology Co., Ltd., Qingdao, China) for stool samples and a direct filtration method for food samples. Briefly, stool specimens were transferred into a 4-ml enrichment buffer, which was provided in the kit. The principal component of the enrichment buffer was the modified Preston broth containing vancomycin, trimethoprim, and amphotericin B, as described in the manual book. The enriched suspension from stool samples was incubated for 24–48 h at 37°C in a microaerophilic atmosphere consisting of 5% O_2_, 10% CO_2_, and 85% N2. Subsequently, 300 μl of cultured enrichment suspension was spotted on Karmali and Columbia agar with a 0.45-μm cellulose membrane filter. At the same time, food samples were fully washed with buffered peptone water (100 ml per 250-g sample), which was concentrated by centrifugation at a low speed of 1,500 g for 15 min. Then, 300–500 ml of concentrated suspension was spotted on Karmali and Columbia agar with a 0.45-μm cellulose membrane filter. After air-drying for 40 min in a biological safety cabinet, the filter membrane was removed and the plates were incubated in an aerobic atmosphere at 30°C for 48 h.

After incubation, small, round, and whitish colonies 2 mm in diameter were plated and confirmed by Gram staining, matrix-assisted laser desorption/ionization time-of-flight mass spectrometry, and real-time polymerase chain reaction (PCR). Mass spectrometry was performed using Flexcontrol software, and the results were interpreted with IVD MALDI Biotyper 2.3 software (Bruker Daltonik GmbH, Bremen, Germany). The criteria for determining the genus and species of bacteria were as follows: 2.300–3.000 points indicated reliable identification to the species level and 2.000–2.299 points indicated reliable identification to the genus level and possible identification to the species level. In this study, scores≥2.000 were considered credible. For PCR identification, a loop was used to collect suspected pure culture colonies, which were resuspended in 200 μl of ultrapure water, boiled for 10 min, and centrifuged for 10 min at 8,000 × g. Subsequently, the supernatant was removed for PCR species identification by a realtime PCR kit (MABSKY BIO-TECH CO., LTD, Shenzhen, China). PCR amplification conditions were as follows: initial denaturation at 94°C for 5 min, followed by 45 cycles of 94°C for 15 s and 60°C for 1 min.

### Antimicrobial susceptibility testing

Antimicrobial susceptibility testing was performed using an agar dilution method-based kit (ZC-CAMPY-013, Zhongchuang Biotechnology Ltd. Corp., Qingdao, China). The test was performed two times in parallel. Mueller-Hinton agar containing 11 different antibiotics was coated onto wells in a 96-well-plate to obtain the MIC of 92 *Arcobacter* strains. The cutoff criteria for each antibiotic were based on the National Antimicrobial Resistance Monitoring System (NARMS-2015: https://www.cdc.gov/narms/pdf/2015-NARMS-Annual-Report-cleared_508.pdf) for *Campylobacter jejuni* and included clindamycin (≥ 1 μg ml^−1^), telithromycin (≥ 8 μg ml^−1^), tetracycline (≥ 2 μg ml^−1^), florfenicol (≥ 8 μg ml^−1^), gentamycin (≥ 4 μg ml^−1^), ciprofloxacin (≥ 1 μg ml^−1^), nalidixic acid (≥ 32 μg ml^−1^), azithromycin (≥ 0.5 μg ml^−1^), and erythromycin (≥ 8 μg ml^−1^), combined with MIC EUCAST (https://mic.eucast.org/search/?search%5Bmethod%5D=mic&search%5Bantibiotic%5D=-1&search%5Bspecies%5D=100&search%5Bdisk_content%5D=-1&search%5Blimit%5D=50:) streptomycin (≥ 4 μg ml^−1^) and chloramphenicol (≥ 32 μg ml^−1^). The quality control bacterial strain was *C. jejuni* ATCC 33560. In addition, we defined multidrug resistance (MDR) as resistance to ≥ 3 classes of antibiotics.

### Extraction of deoxyribonucleic acid and WGS

Deoxyribonucleic acid (DNA) was extracted from Arcobacter isolates. One or two Arcobacter plates from blood agar plates (Huaikai biology, Guangzhou, China) were needed to obtain sufficient material for DNA preparation. Colonies were harvested using fiber swabs and resuspended in 1 ml of phosphate-buffered saline (PBS). The tubes were centrifuged at 16,000 × *g* for 6 min, and the supernatant was discarded. The resulting pellet was further processed for DNA recovery using the bacterial genomic DNA Extraction kit (T132, Tianlong, Shanxi, China) according to the manufacturer's instructions. The concentration of the double-stranded DNA (dsDNA) was examined using a Microplate spectrophotometer (Epoch, Berten Instruments Co., Ltd., Montigny-le-Bretonneux, France).

Next-generation sequencing was performed using the Illumina NovaSeq PE150 (Illumina, San Diego, CA, USA) by Novo Source Technology Co., Ltd., (Beijing, China) and BGI Genomics Co., Ltd., (Beijing, China). To sequence the genomes, a 270-bp paired-end library was constructed and then 150-bp reads were generated. FastQC v0.11.8 (http://www.bioinformatics.babraham.ac.uk/projects/fastqc/) and fastp v0.20.0 (https://github.com/OpenGene/fastp) software tools were applied to evaluate and improve the quality of the raw sequence data, respectively. Low-quality reads were removed in case of the quality scores of ≥3 consecutive bases ≤ Q30. The clean reads were assembled using SOAPdenovo v2.04 (http://soap.genomics.org.cn/soapdenovo.html) and spades v3.13.1 software (Prjibelski et al., 2020). Finally, the assembled sequences were subjected to gene prediction and functional annotation using the Prokka pipeline (Seemann, 2014) and glimmer software (http://ccb.jhu.edu/software/glimmer/index.shtml). The Kyoto Encyclopedia of Genes and Genomes (KEGG) and Clusters of Orthologous Groups (COG) databases were used for functional classification.

### Bioinformatic analyses

The presence of virulence genes was assessed by submitting the assembled genomes to the virulence factor database (VFDB) (http://www.mgc.ac.cn/cgi-bin/VFs/v5/main.cgi?func=VFanalyzer). ResFinder (http://cge.cbs.dtu.dk/services/ResFinder/), and Abricate software (https://github.com/tseemann/abricate), and the Comprehensive Antibiotic Resistance Database (CARD) (https://card.mcmaster.ca/?q=CARD/ontology/35506) were used to predict resistance genes, with a cutoff comprising an *E*-value of at least 1e−10. Cutoffs for identification and query coverage values were >80 and >60%, respectively. Individual missense mutations in *gyrA* conferring ciprofloxacin resistance were detected using BLASTn. Databases including KEGG, COG, SwissProt, and PHI, were used to functionally annotate and classify protein sequences. We extracted the nucleotide sequences of all annotated resistance genes from the genomes, and the genoPlotR package was used to visualize related gene clusters. Based on a previous study (Parisi et al., 2019), 10 common virulence factors (*iroE, irgA, tlyA, pldA, mviN, hecB, hecA, ciaB, cj1349, ciaB, and cadF*) were analyzed using the BLASTn method.

Phylogenetic tree analysis was performed on 177 *Arcobacter* strains, including 85 *Arcobacter* strain sequences (45 *A. butzleri*, 26 *A. cryaerophilus*, and *14 A. skirrowii*), which were downloaded from the GenBank database and 92 strains obtained in this study. Core single-nucleotide polymorphisms (SNPs) were called using Snippy 4.3.6 software (https://github.com/tseemann/snippy) with default parameters and ICDCAB83 as the reference genome. Gubbins software (Croucher et al., 2015) was used as a recombination-removal tool to gain pure SNPs without recombination. Phylogeny reconstruction was performed using the maximum likelihood (ML) method in MEGA 7 software (Kumar et al., 2016) with 1,000 bootstraps.

### Data analysis

SPSS 26.0 (IBM Corp., Armonk, NY, USA) was used for statistical analysis, and the chi-squared test (χ^2^) was used to compare count data between groups. A statistical probability of <0.05 (*p* < 0.05) indicated a statistically significant difference.

## Results

### The prevalence of *Arcobacter* spp. isolated from patients with diarrhea, poultry, beef, pork, vegetables, and seafood

**Figure 1:**
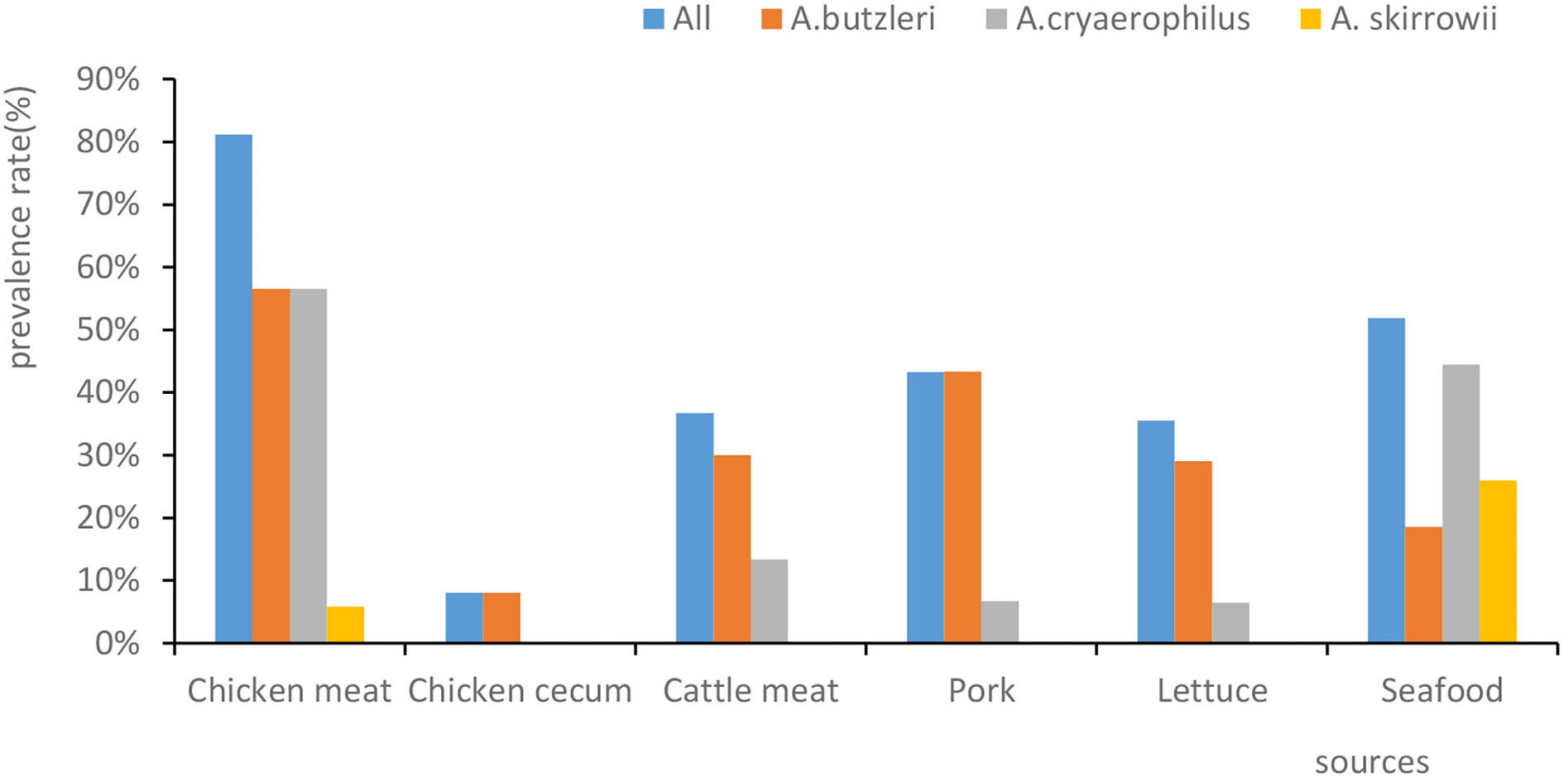
The prevalence rates of *Arcobacter* spp. isolated from different sources. The *x*-axis represents the sources. The *y*-axis represents the prevalence percentage. The color of the columns corresponds to the organisms.

Out of 159 human fecal samples, no *Arcobacter* strains were isolated. In total, 109 *Arcobacter* strains were separated from chicken meat, chicken cecum, beef, pork, lettuce, and seafood (Figure 1). *A. butzleri* was the most prevalent species (33.3%, 79/237), followed by *A. cryaerophilus* (24.9%, 59/237) and *A. skirrowii* (4.6%, 11/237). In 37 of the samples, two or three species of Arcobacter were isolated. Moreover, the prevalence percentage of *Arcobacters* spp. in chicken meat, seafood, pork, beef, lettuce, and chicken cecum was 81.2% (56/69), 51.9% (14/27), 43.3% (13/30), 36.7% (11/30), 35.5% (11/31), and 8% (4/50), respectively (Figure 1). Significantly, *Arcobacter* spp. isolated from chicken cecum (8%, 4/50) had a lower prevalence than those isolated from poultry meat (81.2%, 56/69) (χ^2^ = 10.632, *p* = 0.001).

### Whole genome sequencing

A total of 92 strains (51 *A. butzleri* isolated from 26 chicken, eight beef, seven pork, seven lettuce, and three seafood samples; 40 *A. cryaerophilus* isolated from 29 chicken, three beef, two pork, two lettuce, and four seafood samples; and one *A. skirrowii* isolated from seafood) were characterized by WGS. WGS of Arcobacter isolates were registered in GenBank with accession numbers SAMN30871879 to SAMN30871970. Assemblies consisted of 10–132 contigs. The sequence length was predicted to be between 1,827,334 and 2,453,640 concordant bases. The estimated sizes of the genomes of the 92 strains varied from 1.82 to 2.34 Mb. The guanine-cytosine (GC) content was determined to be 27.78% for *A. skirrowii*, 27.15–27.47% for *A. cryaerophilus*, and 26.65–27.02% for *A. butzleri*.

### Virulence genes and secretion systems

**Figure 2:**
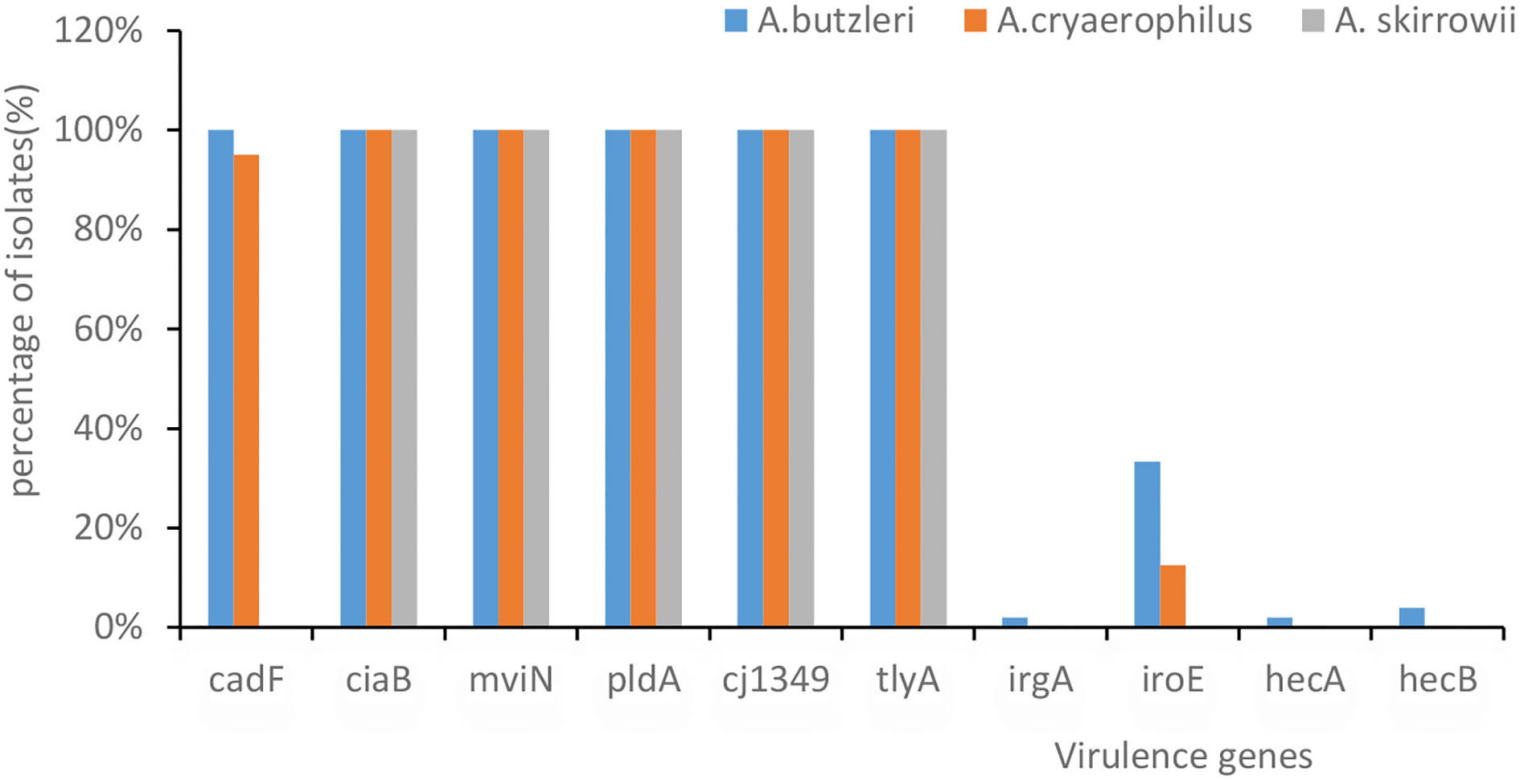
The presence of virulence-associated genes in *Arcobacter* spp. The *x*-axis represents the virulence genes. The *y*-axis represents their percentage of isolates. The color of the columns corresponds to the organisms.

Genome sequencing in VFDB showed that all *A. butzleri* and *A. cryaerophilus* isolates had the following virulence factor genes: *tlyA, mviN, cj1349, ciaB*, and *pldA*, followed by *cadF* (95.7%), *iroE* (23.9%), *hecB (*2.2%*), hecA*, and *irgA* (1.1%) (Figure 2). Moreover, there was no difference in the distribution between *A. butzleri* and *A. cryaerophilus*. Almost no *A. cryaerophilus* strains carried the *IrgA, hecA*, and *hecB* genes. None of the strains had a secretion system. *A. skirrowii* contained the virulence factor genes *tlyA, mviN, cj1349, ciaB*, and *pldA*.

### Genetic prediction of antibiotic resistance and concordance with resistance phenotypes

**Figure 3:**
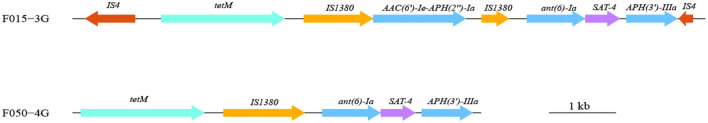
Gene cluster composition in one *A. butzleri* (F050-4G) and one *A. cryaerophilus* (F015-3G) isolate. Different colors represent different genes or categories of the resistance gene. The length and direction of the arrows indicate the size and direction of genes.

All 51 strains of *A. butzleri* contained β-lactam antibiotic resistance genes (*bla*_*OXA*464_or *bla*_*OXA*491_), among which 27 strains contained complete genes and the remaining 24 strains contained incomplete genes. Five strains (F034-1G, F050-4G, F061-2G, F101-1G, and F114.2G) contained three tetracycline resistance genes (*tet (L), tet (H)*, and tet *(M)*). F061-2G contained a macrolide resistance gene (*ere (A)*). F050-4G, which was isolated from pork, contained aminoglycoside resistance genes (*APH (3 ') -IIIa* and *ant (6) -Ia*), streptomycin resistance genes (*SAT-4*), and tetracycline resistance genes (*tet (M)*). These genes might exist as gene clusters in bacteria. The gene cluster size was 6,072 bp, with a GC content of 37.17%, and comprised five genes, including four resistance genes (Figure 3).

Seventeen *A. cryaerophilus* strains contained β-lactam resistance genes, and three of them (F015-3G, F035-7G, and F132-4G) contained tetracycline resistance genes, *tet* (Y), *tet* (H), and *tet* (M). Strain F015-3G isolated from chicken contained one MDR gene island flanked by the insertion sequence IS4 (Figure 3). The size of this MDR gene island was 9,409 bp in length and had a GC content of 33.45%. Seven resistance genes, including three aminoglycosides resistance genes, one tetracycline resistance gene, one streptomycin resistance gene, and two insertion sequences (IS1380), were harbored on this island (Figure 3). This resistance island of F015-3G was similar to that carried by *Campylobacter coli* SH96 (Sequence ID: **MT107516.1**).

One strain of *A. skirrowii* (F198-3G) did not contain resistance genes. Among 51 *A. butzleri* isolates from five sources, streptomycin had the highest resistance rate (98.1%), followed by clindamycin (94.1%), tetracycline (64.7%), azithromycin (43.1%), nalidixic acid (33.4%), and ciprofloxacin (31.3%); others were <10% (Table 1). Among 40 *A. cryaerophilus* isolates from five sources, clindamycin resistance was the highest (90%), followed by streptomycin (70%), tetracycline (52.5%), nalidixic acid, and ciprofloxacin (35%); others were <8% (Table 2). In terms of MDR, 33 *A. butzleri* strains and 23 *A. cryaerophilus* strains were resistant to three or more classes of antibiotics.

**Table 1:** Minimum inhibitory concentrations (MICs) of antimicrobial agents toward 51 *A. butzleri* isolates.

**CLSI antimicrobial class^†^**	**Antimicrobial agent**	**Percentage of all isolates with MIC(**μ**g/ml)**									
		** <0.25**	** <0.5**	**0.5**	**1**	**2**	**4**	**8**	**16**	**≥32**	**≥64**
Aminoglycosides	Gentamicin		11.8		58.8	27.5	2.0				
	Streptomycin					2.0	54.9	39.2		2.0	2.0
Ketolide	Telithromycin	7.8		17.6	21.6	37.3	7.8	5.9		2.0	
Macrolides	Azithromycin		56.9		5.9	7.8	9.8	13.7	3.9		2.0
	Erythromycin		23.5		27.5	35.3	7.8	3.9			2.0
Quinolones	Ciprofloxacin		68.6		2.0		3.9	9.8	7.8	3.9	3.9
	Nalidixic acid						23.5	33.3	9.8	5.9	27.5
Lincosamides	Clindamycin			5.9	49.0	35.3	7.8		2.0		
Phenicols	Florfenicol				3.9	47.1	39.2	7.8	2.0		
	Chloramphenicol		2.0			17.6	58.8	17.6	3.9		
Tetracyclines	Tetracycline				35.3	27.5	23.5	9.8	3.9		

† Clinical and laboratory standards institute.

Gray shadings represent the percentage of resistance isolates.

**Table 2:** MICs of antimicrobial agents to 40 *A. cryaerophilus* isolates.

**CLSI antimicrobial class^†^**	**Antimicrobial agent**	**Percentage of all isolates with MIC(**μ**g/ml)**									
		** <0.25**	** <0.5**	**0.50**	**1**	**2**	**4**	**8**	**16**	**≥32**	**> 64**
Aminoglycosides	Gentamicin		40.0		32.5	25.0					2.5
	Streptomycin		2.5			27.5	35.0	27.5	2.5		5.0
Ketolide	Telithromycin	25.0	2.5	10.0	30.0	27.5	5.0				
Macrolides	Azithromycin		85.0	2.5	5.0	5.0	2.5				
	Erythromycin		42.5		32.5	22.5	2.5				
Quinolones	Ciprofloxacin		65.0		2.5			5.0	17.5	10.0	
	Nalidixic acid						15.0	40.0	10.0	2.5	32.5
Lincosamides	Clindamycin	5.0		5.0	30.0	50.0	7.5		2.5		
Phenicols	Florfenicol				7.5	45.0	40.0	7.5			
	Chloramphenicol				2.5	30.0	60.0	5.0	2.5		
Tetracyclines	Tetracycline		12.5		35.0	25.0	22.5		5.0		

† Clinical and laboratory standards institute.

Gray shadings represent the percentage of resistance isolates.

Five *A. butzleri* isolates, including tetracycline resistance genes (*tet (L), tet (H)*, and *tet (M)*), were phenotypically resistant to tetracycline. Thirteen *A. butzleri* isolates with *gyrA* (C254T) mutation were phenotypically resistant to ciprofloxacin. One of the *A. butzleri* isolates carried the *ere(A)* gene, which is associated with erythromycin resistance (MIC value ≥ 64 μg/ml). One *A. butzleri* isolate (F050-4G) isolated from pork had *gyrA* (C254T) mutation, contained a streptomycin resistance gene (*Sat-4*), a tetracycline resistance gene (*tet (M)*) and aminoglycoside resistance genes (*APH (3 ') -IIIa* and *ant (6) -Ia*), and was phenotypically resistant to ciprofloxacin, streptomycin, and tetracycline.

Three *A. cryaerophilus* isolates containing tetracycline resistance genes (*tet (L), tet (H)*, and *tet (M)*) were phenotypically resistant to tetracycline. Fourteen *A. cryaerophilus* isolates with a *gyrA* (C254T) mutation were phenotypically resistant to ciprofloxacin. One *A. cryaerophilus* isolate (F015-3G) isolated from chicken had a *gyrA* (C254T) mutation, contained a streptomycin resistance gene (*Sat-4*), a tetracycline resistance gene (*tet (M)*), and aminoglycoside resistance genes (APH (3 ') -IIIa and ant (6) -Ia), and showed resistance to ciprofloxacin, streptomycin, and tetracycline.

### Phylogenetic reconstruction

**Figure 4:**
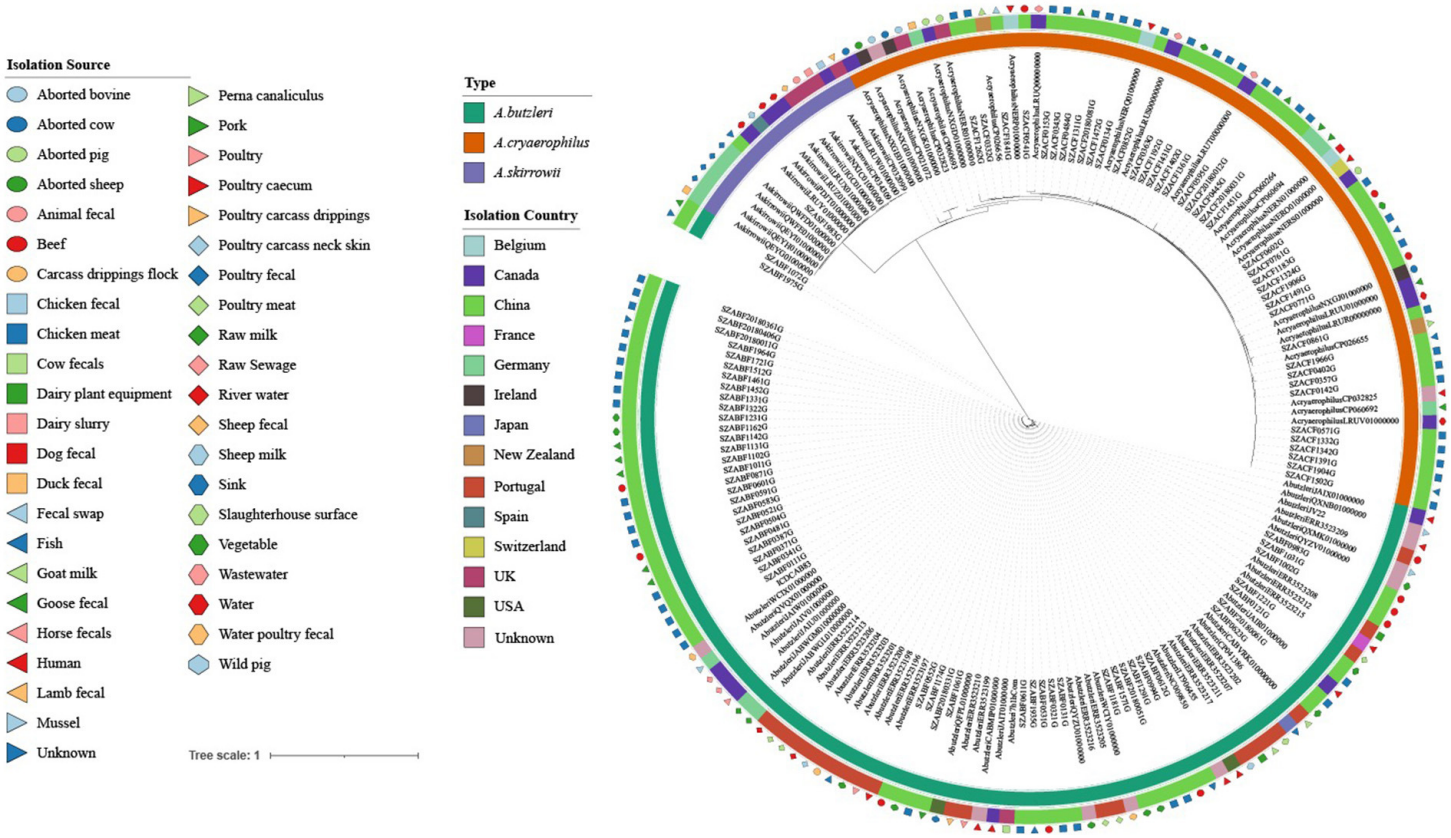
Maximum likelihood (ML) tree of the core genome alignments of strains isolated from various sources and countries with 1,000 bootstraps. Different colors and shapes indicate various sources. From the inner circle to the outer circle, strains, Type, Isolation Country, and Isolation Source are listed.

Phylogenetic tree analysis revealed that the 177 strains were mainly divided into three groups (*A. butzleri, A. cryaerophilus, and A. skirrowii*). There was no obvious aggregation phenomenon in each group according to the source of the host or the sampling site, indicating that *Arcobacter* strains showed high genetic diversity (Figure 4).

## Discussion

*Arcobacter* spp. is globally recognized as one of the causes of acute gastroenteritis (Zhang et al., 2019; Brückner et al., 2020). The main transmission source of *Arcobacter* spp. in humans is *via* the consumption of contaminated water and food (Collado and Figueras, 2011; Zambri et al., 2019). This study provides updated information on the incidence, genetic diversity, and antimicrobial susceptibility of *Arcobacter* spp. Samples were collected from various sources in Shenzhen, China.

Our results showed that the prevalence of *Arcobacter* spp. ranged from high to low in chicken meat (81.2%, 56/69), seafood (51.9%, 14/27), pork (43.3%, 13/30), beef (36.7%, 11/30), lettuce (35.5%,11/31), chicken cecum (8.0%, 4/50), and human feces (0.0%,0/159). The prevalence of *Arcobacter* spp. in human feces ranged from 0.2 to 3.6% in studies conducted in Germany, Chile, Portugal, India, and Turkey (Patyal et al., 2011; Kayman et al., 2012; Ferreira et al., 2014; Fernandez et al., 2015; Brückner et al., 2020), which was somewhat consistent with our result that *Arcobacter* spp. was not isolated from any of the 159 fecal samples. In contrast to our findings, the prevalence of *Arcobacter* isolated from chicken meat was higher than the prevalence found in Iran (26%) (Khodamoradi and Abiri, 2020), Germany (26.8%) (Lehmann et al., 2015), and Tunisia (13.42%) (Jribi et al., 2020), but similar to that found in Beijing (73.33%) (Wang et al., 2016). Jasim et al. (2021) reported that (120/1,293) the prevalence of positive beef samples in Iraq was 9.25%, which was lower than that in the present study. Mottola et al. (2021) reported that its prevalence in ready-to-eat vegetables was 14.5% (16/110), which was lower than that in the present study. Zhang et al. (2019) reported that the contamination rate for seafood was 17.6% (56/318), which was lower than that reported here.

Furthermore, our results showed a lower prevalence of *Arcobacter* spp. in the chicken cecum (8%, 4/50) than in chicken meat (81.2%, 56/69) (χ^2^ = 62.073, *p* = 0.000), which was possibly due to cross-contamination of meat in both the slaughter and retail market environments.

Barboza et al. (2017) reported that the prevalence of chicken cecal content was 5.26% (8/152). Schönknecht et al. (2020) reported that the prevalence of *Arcobacter* spp. in the chicken cecum was 3% (1/29), which was lower than other intestinal contents. Cecal contents might not be the main *Arcobacter* reservoir inside chickens. The prevalence of *Arcobacter* spp. isolated from various sources in this study might change with seasons and climate. We will continue to conduct relevant pathogen monitoring research in the future.

In the current study, almost all strains of *Arcobacter spp*. have β-lactam antibiotic resistance genes. It has been speculated that *Arcobacter* spp. may be resistant to β-lactamase. Several studies reported that β-lactam resistance may be caused by the presence of three putative β-lactamases (AB0578, AB1306, and AB1486) identified in the RM4018 genome, which are enhanced by the occurrence of the lrgAB operon (ab0179 and ab0180) and may regulate tolerance to penicillin in *Staphylococcus* (Bayles, 2000; Groicher et al., 2000; Miller et al., 2007). However, we did not perform an experiment showing the phenotypic resistance of *Arcobacter* spp. to β-lactam antibiotics. Therefore, further investigation on the resistance phenotype and mechanism of β-lactam antibiotics is needed. For severe clinical diseases caused by *Arcobacter* spp., fluoroquinolones, tetracyclines, macrolides, and aminoglycosides are recommended for treatment (Ferreira et al., 2016). A meta-analysis of *Arcobacter* spp. antibiotic resistance in 2019 (Ferreira et al., 2019) reported that fluoroquinolone resistance ranged from 4.3 to 14.0%, whereas it was 0.8–7.1% for tetracyclines, 10.7–39.8% for macrolides, and 1.8–12.9% for aminoglycosides. In this study, the resistance rates of A. *butzleri* and A. *cryaerophilus* to nalidixic acid (33.4%, 35%), ciprofloxacin (31.3%, 35%), and tetracycline (64.7%, 52.5%) were much higher than previously reported (Ferreira et al., 2019). However, Jribi et al. (2020) reported a high level of resistance to tetracycline (100%) in *Arcobacter* spp. isolated from poultry in Tunisia.

The resistance rates of 51 *A. butzleri* isolates to erythromycin, azithromycin, and telithromycin were 5.9, 43.1, and 7.9%, respectively. All *A. cryaerophilus* isolates were completely sensitive to erythromycin. The resistance rate of *Arcobacter* spp. to erythromycin is consistent with a previous study (3.6%, 3/84) (Kietsiri et al., 2021). Moreover, streptomycin resistance rates for *A. butzleri* and *A. cryaerophilus* were 41.2 and 32.5%, respectively, which were higher than those reported in a previous study (Ferreira et al., 2016, 2019). Therefore, further attention should be paid to streptomycin resistance.

One strain each of *A. butzleri* (F050-4G) and *A. cryaerophilus* (F015-3G) contained resistance island gene clusters, which contained multiple antibiotic resistance genes and were located near the transposon. The resistance island in *A. cryaerophilus* (F015-3G) was inserted into IS4. The GC content of the two resistance islands was significantly higher than that of the genome of *Arcobacter* spp. We suspected that the resistance island might have been obtained by the horizontal gene transfer. Several studies reported that the use of antibiotics in animals might cause MDR and then transfer to humans (Chang et al., 2015; Dekker et al., 2019).

Several studies (Webb et al., 2018; Hodges et al., 2021) showed that the base mutation in the *gyrA* gene was associated with a higher level of resistance to ciprofloxacin. In our study, 13 *A. butzleri* and 14 *A. cryaerophilus* isolates with *gyrA* (C254T) mutation were 100% phenotypically resistant to ciprofloxacin. Moreover, the genes *aph (3 ') -IIIa* and *ant (6) –Ia* were reported to correlate with resistance to kanamycin and streptomycin, respectively (Ntilde et al., 2018; Cho et al., 2020), and in our study, two isolates carried the ant (6) –Ia gene and were phenotypically resistant to streptomycin. In addition, isolates that carried tetracycline resistance genes were phenotypically resistant to tetracycline, whereas the occurrence of *ere(A)* gene was associated with erythromycin resistance, which was consistent with previous studies (Gao et al., 2015; Zhao et al., 2016; Webb et al., 2018).

The number of antibiotic resistance genes varied greatly between the isolates. Some isolates harbored multiple antibiotic resistance genes, and specific resistance genes were detected in the corresponding antibiotic resistance isolates. Strains containing resistance genes were resistant to the corresponding antibiotics or had higher MIC values. A C254T mutation was found in some strains, resulting in a Thr to Ile substitution at position 85 of the deduced protein sequence. This substitution in *A. butzleri* and *A. cryaerophilus* isolates could be responsible for the observed fluoroquinolone resistance. A C254T mutation in *gyrA*, which resulted in a Thr to Ile substitution in *gyrA* were found in all ciprofloxacin resistance strains. This substitution in *A. butzleri* and *A. cryaerophilus* isolates could be responsible for the observed fluoroquinolone resistance.

*Arcobacter skirrowii, A. cryaerophilus*, and *A. butzleri* isolated from food and originating from animals commonly carry *tlyA, pldA, mviN, ciaB, cj1349*, and *cadF* (Douidah et al., 2012; Khoshbakht et al., 2014; Parisi et al., 2019; Khodamoradi and Abiri, 2020). Rathlavath et al. (2017) reported that the majority of *A. butzleri* isolated from seafood and the coastal environment contained six common virulence genes [cadF (89.7%), cj1349 (97.2%), ciaB (95.9%), mviN (100%), pldA (91.1%), and tlyA (91.8%)] but relatively lower amounts of *hecA* (10.8%), *hecB* (19%), *iroE* (12.9%), and *irgA* (17.6%). Similarly, our study found that more than 90% *Arcobacter* contained these six common virulence factors. It was found that different virulence genes had different functions, e.g., *tlyA* encoding hemolysin and *pldA* encoding the outer membrane phospholipase A are involved in erythrocyte lysis; *mviN* is required for the biosynthesis of peptidoglycan; *ciaB* is required for the biosynthesis of peptidoglycan; and both *cadF* and *cj1349* encode fibronectin-binding protein (Parisi et al., 2019). *A. butzleri* and *A. cryaerophilus* showed no differences in the distribution of these virulence factors, which was in contrast to the results of a previous study (Sekhar et al., 2017) in which *A. butzleri* was observed t*o* carry more of these virulence factors than *A. cryaerophilus*.

In addition to poultry, vegetables and seafood are also important transmission routes for *Arcobacter* infection in humans. The resistance island gene cluster found in pork and chicken meat and the carriage of virulence factors could be a potential health risk to human health.

## Author's note

The first author, YM MD, female, was born in 1991, whose specialty is pathogenic microbiology; CJ, female, was born in 1982, majoring in analysis of genetic characteristics of *Campylobacter* and *Arcobacter*.

## Data availability statement

The data presented in the study are deposited in the GenBank with accession numbers SAMN30871879 to SAMN30871970.

## Author contributions

YD and MZ designed the experiments. YM, CJ, MY, HC, and JH participated in the sample collection and performed the experiments. GZ performed the genome bioinformatic analysis. YM and CJ wrote this paper. All authors read and approved the submitted manuscript.

## Funding

This work was supported by the Sanming Project of Medicine in Shenzhen, China (SZSM201803081) and the Key Discipline Construction Subsidy of Nanshan District, Shenzhen, China.

## Conflict of interest

The authors declare that the research was conducted in the absence of any commercial or financial relationships that could be construed as a potential conflict of interest.

## Publisher's note

All claims expressed in this article are solely those of the authors and do not necessarily represent those of their affiliated organizations, or those of the publisher, the editors and the reviewers. Any product that may be evaluated in this article, or claim that may be made by its manufacturer, is not guaranteed or endorsed by the publisher.
